# Multivariate Analysis of Evoked Responses during the Rubber Hand Illusion Suggests a Temporal Parcellation into Manipulation and Illusion-Specific Correlates

**DOI:** 10.1523/ENEURO.0355-21.2021

**Published:** 2022-01-07

**Authors:** Placido Sciortino, Christoph Kayser

**Affiliations:** Department for Cognitive Neuroscience, Faculty of Biology, Bielefeld University, Universitätsstr. 25, 33615 Bielefeld, Germany

**Keywords:** body consciousness, body ownership, EEG, rubber hand, rubber hand illusion, self-consciousness

## Abstract

The neurophysiological processes reflecting body illusions such as the rubber hand remain debated. Previous studies investigating the neural responses evoked by the illusion-inducing stimulation have provided diverging reports as to when these responses reflect the illusory state of the artificial limb becoming embodied. One reason for these diverging reports may be that different studies contrasted different experimental conditions to isolate potential correlates of the illusion, but individual contrasts may reflect multiple facets of the adopted experimental paradigm and not just the illusory state. To resolve these controversies, we recorded EEG responses in human participants and combined multivariate (cross-)classification with multiple Illusion and non-Illusion conditions. These conditions were designed to probe for markers of the illusory state that generalize across the spatial arrangements of limbs or the specific nature of the control object (a rubber hand or participant’s real hand), hence which are independent of the precise experimental conditions used as contrast for the illusion. Our results reveal a parcellation of evoked responses into a temporal sequence of events. Around 125 and 275 ms following stimulus onset, the neurophysiological signals reliably differentiate the illusory state from non-Illusion epochs. These results consolidate previous work by demonstrating multiple neurophysiological correlates of the rubber hand illusion and illustrate how multivariate approaches can help pinpointing those that are independent of the precise experimental configuration used to induce the illusion.

## Significance Statement

The neurophysiological signatures of body illusions such as the rubber hand remain debated. To reconcile the fragmented picture painted by previous work, we capitalized on a representation-centered approach to analyze human EEG recordings using multivariate classification and designed our study around two experimental Illusion conditions and multiple non-Illusion conditions that varied the relative hand position, or the nature of the control object. Our results show illusion-specific activations early after stimulus onset and during a prolonged time window, thereby consolidating the fragmented picture in the literature.

## Introduction

The neurophysiological processes underlying multisensory body perception and the sense of body ownership are often studied using illusions such as the rubber hand (Botvinick and Cohen, 1998; Blanke, 2012; Longo and Haggard, 2012; Riemer et al., 2019). There, participants watch an artificial hand being stimulated in synchrony with their own occluded hand, which results in the illusory experience of the rubber hand becoming embodied (Tsakiris and Haggard, 2005; Blanke, 2012). Despite many behavioral (Botvinick and Cohen, 1998; Tsakiris and Haggard, 2005; Longo and Haggard, 2012) and neuroimaging studies (Ehrsson et al., 2004, 2005; Petkova et al., 2011; Bekrater-Bodmann et al., 2012; Brozzoli et al., 2012; Gentile et al., 2013; Guterstam et al., 2013; Limanowski and Blankenburg, 2016) involving the rubber hand illusion the underlying electrophysiological correlates, such as studied using EEG, remain debated.

In fact, previous electrophysiological studies disagree on the relevant illusion-specific activations. For example, several studies have investigated the evoked potentials produced by the repetitive visuo-tactile stimulation and asked when and how these responses are affected by the illusory state, that is, the subjective state of the individuals when they experience the rubber hand as becoming part of their own body, an experience that is absent in the control conditions. Some studies reported illusion-correlates only at early (∼50 ms) latencies relative to the stimulation events and advocated for a low-level origin in early somatosensory cortex (Zeller et al., 2015; Sakamoto and Ifuku, 2021). Support for such an origin also comes from studies on the general enhancement of somatosensory processing by multisensory inputs on the viewed body (Cardini et al., 2011, 2012). However, other studies advocated for illusion-correlates only at latencies of around 120–200 ms, possibly related to the detection of mismatching sensory signals in temporo-parietal cortex (Press et al., 2008), while a third group of studies emphasized illusion-related activity at even longer latencies of around 300–400 ms and suggested high-level cognitive processes as underlying sources (Peled et al., 2003; Rao and Kayser, 2017). This body of previous work also diverges on whether perceiving the illusion would enhance or attenuate evoked responses (Zeller et al., 2015), with some studies on related body illusions reporting no localized changes in evoked activity at all (Pyasik et al., 2021).

One reason for these diverging results may be the different experimental settings used to induce the illusion. For example, the illusion has been induced when the real hand is besides (Lloyd, 2007; Kammers et al., 2011; Bekrater-Bodmann et al., 2014; Riemer et al., 2015; Rao and Kayser, 2017) or below the rubber hand (Rohde et al., 2011; Preston, 2013; Zeller et al., 2015), resulting for example in possibly different illusion onset times. Another reason may be that few studies asked which neurophysiological signatures generalize across experimental settings used to isolate the neurophysiological correlates of the illusory state. While individual studies reported correlates at different latencies, it remains unclear at which latencies (early, intermediate, or late) relative to the visuo-tactile stimulation brain activity reliably differentiates the illusory state from multiple and suitable control conditions. This question about neural representations generalizing across experimental contrasts, however, is difficult to address in the classical activation-mapping framework used by previous studies, for two reasons (Kriegeskorte et al., 2006; Hebart and Baker, 2018). First, the analysis of electrode-wise evoked responses implicitly assumes a common reference model of the brain and the same spatial configuration of the relevant illusion-related activations across participants. Yet, the individual variability in brain morphology may invalidate this assumption (Van Horn et al., 2008; Li et al., 2019; Eichert et al., 2020) and one study on bodily illusions provided direct evidence that illusion-specific correlates have been reflected in distributed rather than localized patterns of EEG responses (Aspell et al., 2012). Second, the typical pairwise comparison of experimental conditions makes it difficult to generalize significant differences across multiple experimental conditions, a critical test required to establish which neurophysiological signatures generalize across experimental settings used to induce the illusion (Kriegeskorte et al., 2006; Hebart and Baker, 2018).

In an attempt to address this challenge, the present study employed a linear multivariate classification framework to probe whether and when neurophysiological correlates of the illusory state generalize across distinct illusion-inducing configurations. Such a linear classification framework has been ubiquitously used to uncover the neurophysiological representations of sensory and cognitive processes (Parra et al., 2005; Cichy et al., 2014; Grootswagers et al., 2018; Guggenmos et al., 2018; Keitel et al., 2020), and, rather than constraining the relevant sources in space to be identical across participants, it allows relying on the statistical properties of the data (Parra et al., 2005; Blankertz et al., 2011; Grootswagers et al., 2017, 2018). We here exploit this approach to overcome the two above mentioned problems: it allows us to directly probe whether and when neurophysiological correlates of the illusory state generalize across distinct illusion-inducing configurations, and it allows for distinct spatial patterns of neurophysiological illusion correlates in individual participants.

To this end, we combined EEG recordings in human participants with a computer-controlled setup to induce the illusion, allowing the precise alignment of rapid neurophysiological responses to the experimental stimulation (Rao and Kayser, 2017). Importantly, to address the question of when neurophysiological signatures generalize across experimental conditions, we employed two experimental configurations to induce the illusion (having the rubber and besides or below the participant’s hand), two non-Illusion control conditions (a body-incongruent rubber hand tilted by 90°, and participant’s real hand), and we investigated a contrast devoted to comparing evoked responses within the same experimental Illusion trial, between the period before and after participants reporting the onset of the illusion, a contrast already explored in fMRI and EcoG studies (Ehrsson et al., 2004; Guterstam et al., 2019). We complemented the EEG recordings with measurements of skin conductance, an index of autonomous responses that is sensitive to the embodiment of a rubber hand (Armel and Ramachandran, 2003; Ehrsson et al., 2007; Gentile et al., 2013; D’Alonzo et al., 2020). This allowed us to ask the more explorative question of whether the neurophysiological correlates of the illusory state are related to these bodily correlates.

In sum, by employing a multivariate framework our study aimed to make methodological advances on how the neurophysiological signatures of body illusions can be investigated. Our results provide conceptually novel results by highlighting when in time neurophysiological signatures possibly generalize across experimental conditions, and help to reconcile the diversity of results reported in previous EEG studies on the rubber hand illusion (also abbreviated in RHI from now on).

## Materials and Methods

### Experimental conditions

**Figure 1.**
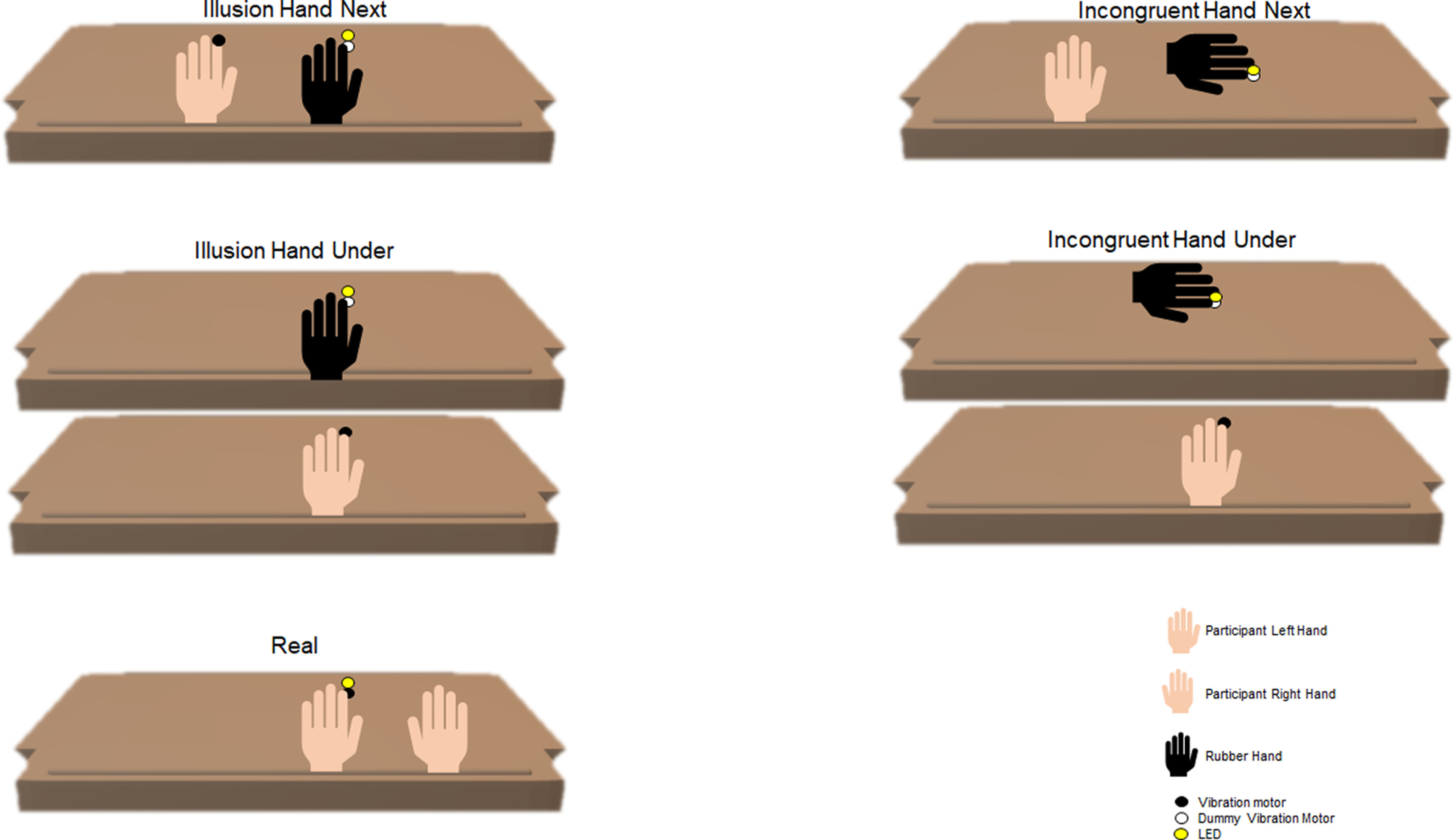
Schematic of the five experimental conditions. The experiment involved two Illusion conditions that differed in the spatial arrangement of the real and artificial limbs (horizontal or vertical displacements), and the two respective Incongruent conditions obtained by rotating the rubber hand into an unphysiological body position by 90°. Finally, we included a Real condition in which the rubber hand was absent and multisensory stimulation was delivered to the participant’s real hand.

Experiments were performed in a darkened and electrically shielded room (Ebox, Desone). Participants sat on a comfortable chair in front of a one-compartment, open-ended box placed on a two-story wooden platform. Five experimental conditions were used (Fig. 1). These differed in the relative position of participant’s hand relative to the rubber hand (besides or below the rubber hand hand), the relative orientation of the rubber hand (rubber hand aligned to the body, or tilted by 90°), or did not involve any rubber hand (with the stimulation on the real hand). Each trial lasted 3 min and consisted of 180 visuo-tactile stimulation events of 100-ms duration and presented with an interstimulus interval (ISI) was 900 ms, resulting in a visuo-tactile stimulation frequency of 1 Hz. Visual stimuli were delivered by white light-emitting diodes (LED; Seeedstudio, 10-mm diameter) and tactile stimuli were delivered to the participant’s fingertip by a vibration motor (Grove: Vibration motor, Seeedstudio). Both stimuli were controlled via MATLAB and two Arduino Uno prototyping platforms. To facilitate the alignment of stimulation events with the EEG data we routed a copy of the voltage controlling the DC motor to an analog input of the EEG system.

The precise experimental conditions were designed as follows. In the Illusion Hand Next condition, a lifelike rubber hand (for men: a silicon cosmetic glove, model 102LS, for women: model 102LS, ORTHO-REHA Neuhof GmbH) was positioned on top of the platform (horizontal plane) in front of the participants in an anatomically congruent orientation, as typically used to induce the illusion. The index finger of the rubber hand was placed on a dummy vibration motor, which did not vibrate. The participant’s left hand was covered with a blanket, hidden to the participant’s view, and was positioned at a distance of 10 cm from the rubber hand in the horizontal plane. The tip of the participant’s index finger was placed on the vibration motor, while the right hand was placed at the other end of the platform in reaching distance of a keyboard. The LED was positioned 5 mm above the dummy motor near the rubber hand. The somatosensory stimulus on the participant’s hand and the synchronous visual stimulus near the rubber hand reliably induce the illusion, as discussed below. In the Incongruent Hand Next condition, the rubber hand was placed in an anatomically incongruent position at a 90°angle. In the Illusion Hand Under condition, the rubber hand was placed in front of the participant, while the participant’s real hand was covered with a blanket and placed in the lower panel of the platform 10 cm under the rubber hand in the vertical plane. Otherwise, the setup was the same as for the Illusion Hand Next condition. In the Incongruent Hand Under condition, the rubber hand was placed in an anatomically incongruent position, at a 90° angle below participants’ hand. We also included a control condition in which the rubber hand was absent. In this Real condition, no rubber hand was present, and the index finger of the left hand was placed on a vibration motor positioned 5 mm below the LED. The right hand was in the same position as in the Illusion and Incongruent conditions. In the following text, we denote with the capitalized Illusion, the respective experimental conditions, while we use the non-capitalized Illusion to refer to the phenomenon in general.

The five experimental conditions were presented in a pseudorandomized order for each participant. Each condition was repeated four times, for a total of 20 trials per participant. The choice of a rotated rubber hand for the Incongruent condition was made based on previous studies showing that an unrealistic posture abolishes the illusion reliably (Pavani et al., 2000; Ehrsson et al., 2004; Rao and Kayser, 2017; Riemer et al., 2019). Importantly, another often used control condition involving the asynchronous stimulation of the real and rubber hands can result in participants also reporting the illusion in presumed control conditions, and makes specific assumptions about the temporal duration of the multisensory binding window, which the Incongruent condition avoids (Valenzuela Moguillansky et al., 2013; Costantini et al., 2016; Fuchs et al., 2016). The Real condition involves the stimulation of the participant’s real hand, while maintaining the same posture and position of the rubber hand, varying only the nature of the embodied limb (rubber hand in the Illusion condition, real hand in the Real condition; Zeller et al., 2015, 2016; Rao and Kayser, 2017). Since the posture and the nature of the stimulation was the same as for the Illusion condition, we hypothesized that any change between the Illusion and the Real should reflect the illusory feeling and the nature of the embodied object, a dimension not probed by the misalignment of any real and artificial hands or by the asynchronous stimulation of the artificial hands as has been used in some previous studies.

We chose a computer-controlled stimulation setup using LED stimuli as a previous study has shown that such a setup can reliably induce the RHI (Rao and Kayser, 2017). In particular, the LED and vibration stimulus provide a synchronized reference frame between the participant’s real hand and the rubber hand, which leads to the induction of the illusion (Shimada et al., 2009; Evans and Blanke, 2013; Bekrater-Bodmann et al., 2014; Rao and Kayser, 2017). Indeed, previous work has shown that the RHI can be reliably induced across different setups and manipulations that do not necessarily need to involve the presence of an experimenter stroking both hands with a brush (Bertamini and O’Sullivan, 2014; Kalckert and Ehrsson, 2014; Guterstam et al., 2015; Rao and Kayser, 2017). Furthermore, the brief and temporally precise stimulation provided by LED and the vibration motor allows the precise alignment of rapid neurophysiological signals to the stimulation sequence, which becomes intrinsically difficult with temporally imprecise events such as manual stroking (Rao and Kayser, 2017).

### Participants

To ensure that participants enrolled in the main EEG experiment were indeed able to feel the illusion, we conducted a pretest on participants of either sex. None of the participants tested in the pretest reported having participated in a study involving the rubber hand or similar body illusions before. During the pretest four conditions were presented in pseudo-random order (the two Illusion conditions and their respective Incongruent conditions), each presented once. To probe whether or when participants felt the illusion, we capitalized on question three from the common rubber-hand questionnaire (Table 1; Botvinick and Cohen, 1998). Specifically, we instructed participants to press (using their right hand) a key on a computer keyboard when they were “feeling the rubber hand as belonging to their body.” After each trial, they were asked to also verbally confirm that this statement applied. For the main study we invited only participants who in the pretest had indicated feeling the illusion in both Illusion conditions and who did not report feeling the illusion in any Incongruent condition. The criteria to be included in the main study were reporting the illusion onset with a button press, a mean positive score for the three illusion statements and a mean negative score for the control statements (Botvinick and Cohen, 1998). A total number of 24 healthy right-handed participants (9 males, 15 females) participated in this study were included in the main study. That only about half the pretested naive participants reported feeling the illusion in the present experiment is largely in line with previous work, where reported numbers vary between half and two-thirds (Ehrsson et al., 2004, 2007; Lloyd, 2007; Rao and Kayser, 2017; Riemer et al., 2019; Reader et al., 2021, p 202). All participants gave written informed consent before participation in accordance with the Declaration of Helsinki. All protocols conducted in this study were approved by the Ethics Committee of Author University.

**Table 1:** Questionnaire used to assess the subjective feeling of the illusion (adapted from Botvinick and Cohen, 1998**)**

1	During the last trial it seemed as I were feeling the touch in the location where I saw the rubber hand touched	Illusion
2	During the last trial it seemed as though the touch I felt was caused by the vibration motor under the rubber hand	Illusion
3	During the last trial I felt as if the rubber hand were my hand	Illusion
4	During the last trial it felt as my (real) hand was drifting towards the rubber hand	Control
5	During the last trial it seemed as I might have more than one left hand or arm	Control
6	During the last trial it seemed as if the touch I was feeling came from somewhere between my own hand and the rubber hand	Control
7	During the last trial I felt as my (real) hand was turning rubbery	Control
8	During the trial it appeared (visually) as if the rubber hand were drifting towards left (towards my hand)	Control
9	During the trial the rubber hand began to resemble to my own hand in term of shape, skin tones, freckles or some other features	Control

The questionnaire included nine statements describing the underlined phenomena. Participants indicated their response on a 7-scale ranging from “strongly agree” (−3) to “strongly disagree” (+3).

The main experiment took place on a different day than the pretest. During the main experiment four repeats of each of the five conditions were administered in pseudo-random order for each participant, hence comprising 20 trials in total. Each condition lasted for 3 min of visuo-tactile stimulation (180 stimulation events). Because administering the rubber hand questionnaire 20 times is unlikely to yield sensitive results, we capitalized on the button press response as a practical and brief implementation of a test of the main item of the questionnaire, i.e., question three on the embodiment of the rubber hand. We hence relied on this as an index of whether and when during each trial participants started to feel the illusion. We also administered the full questionnaire (Table 1; Botvinick and Cohen, 1998) at the end of the first repeat of each of the two Illusion conditions. From these we calculated the average scores for illusion (Illusion Hand Next: 2.38 ± 0.53, mean ± SEM; Illusion hand under: 2.61 ± 0.53) and control statements (hand next: −1.47 ± 0.76; hand under: −1.56 ± 0.80), which suggest that participants were indeed feeling the illusion. One participant was excluded from the main study as this participant reported feeling the illusion also during one Incongruent trial and one participant was excluded because of reporting the illusion for only one of the two Illusion conditions. Hence, we report EEG data for an *n* of 22.

#### EEG recording and preprocessing

EEG signals were continuously recorded using a 128 channel BioSemi (BioSemi, B.V.) system with Ag-AgCl electrodes mounted on an elastic cap (BioSemi). Four additional electrodes were placed at the outer canthi and below the eyes to obtain the electro-oculogram (EOG). Electrode offsets were kept below 25 mV. Data were acquired at a sampling rate of 1028 Hz. Data analysis was performed with MATLAB (The MathWorks Inc.) using the FieldTrip toolbox (Oostenveld et al., 2011). Data were bandpass filtered between 0.6 and 90 Hz, and resampled to 200 Hz as in our previous work (Kayser and Kayser, 2020; Park and Kayser, 2021) for further processing. Subsequently, the data were denoised using Independent component analysis (ICA) and components reflecting muscular artefacts, eye blinks, eye movements as well as poor electrode contacts were identified based on recommendations in the literature and confirmed based on visual inspection (O’Beirne and Patuzzi, 1999; Campos Viola et al., 2009; Hipp and Siegel, 2013). Overall, we removed an average of 15.0 ± 1 (mean ± SEM) components per participant, a number comparable to previous studies using a very similar analysis pipeline (Grabot and Kayser, 2020; Bröhl and Kayser, 2021; Park and Kayser, 2021).

For subsequent data analysis, we epoched the data around each visuo-tactile stimulation event, with epochs lasting from −400 ms prestimulus to 400 ms poststimulus onset. This resulted in a total number of 180 epochs for each trial. Epochs were combined within each condition, were bandpass filtered between 1 and 40 Hz (two-pass third order Butterworth filters) using the FieldTrip toolbox, to emphasize within-epoch changes and to remove slow drifts that span multiple epochs (Park and Kayser, 2021). Epochs with signals exceeding a level of 165 μV were considered as artifact and removed. Importantly, to render the main analysis of the Illusion conditions specific to participants experiencing the illusory state, we only included epochs after the time point at which participants indicated the onset of the illusion. Hence for any analysis of the Illusion conditions, we included only epochs after the trial-specific onset of the subjective illusion state. Because this effectively removes the early epochs in each 3-min trial, we applied a similar selection to the other conditions; here, we removed epochs before the participant-specific median reaction time obtained from all Illusion trials. The resulting number of epochs available for each condition for both Illusion conditions was 897 ± 51 (mean ± SEM), for the Incongruent conditions 845 ± 56, and for the Real condition 434 ± 33 epochs. In a separate analysis, we contrasted the epochs of the Illusion trials before the illusion onset with those in the Illusion trials subsequent to the illusion onset.

#### Analysis of EEG activity using single-trial classification

To quantify whether and when EEG activity differed between experimental conditions, we used single-trial classification based on a regularized linear discriminant analysis (LDA; Parra et al., 2005; Blankertz et al., 2011). We used this specific classifier as it has been successfully used over the last decade in conjunction with neuroimaging data and because it has proven equally powerful as computationally more complex algorithms (Grootswagers et al., 2017). As typical for LDA analyses, the epoched data were binned into overlapping time bins: we here relied on bins of 60-ms duration, and the classifier was computed at 10-ms time steps, similar as in our previous work (Park and Kayser, 2021). The analysis thereby emphasizes the low-frequency components of the recorded EEG signals, which have highest signal-to-noise ratio and are less contaminated by muscular or ocular artefacts, in contrast to high frequency activity (Hipp and Siegel, 2013). The data are shown such that the time axis refers to the center of these 60-ms time bins. The regularization parameter was set to 0.1 as in previous work (Park and Kayser, 2019), and we based the classifier on “pseudo-trials” obtained from four epochs. For each participant, the classifier performance was obtained as the receiver operating characteristic area under curve (AUC), computed from sixfold cross-validation (preliminary tests had revealed that using more cross-validation folds provides no benefit to the results). That is, we trained the data on five-sixth of the data and tested the classification performance on the remaining one-sixth of the data. Because the available epoch numbers differed across conditions, we relied on a re-sampling procedure: classification performance was derived by randomly selecting equal numbers of epochs for each contrast (randomly selecting a number of trials that corresponds to 80% of the smaller number of available epochs for each condition, and averaging the classification performance over 50 repeats of this procedure). We derived participant-wise scalp topographies for each classifier by estimating the corresponding forward model, defined as the normalized correlation between the discriminant component and the EEG activity (Parra et al., 2005). The group-averaged forward models at time points of interest are shown as insets in Figures 4, 5.

**Figure 2.**
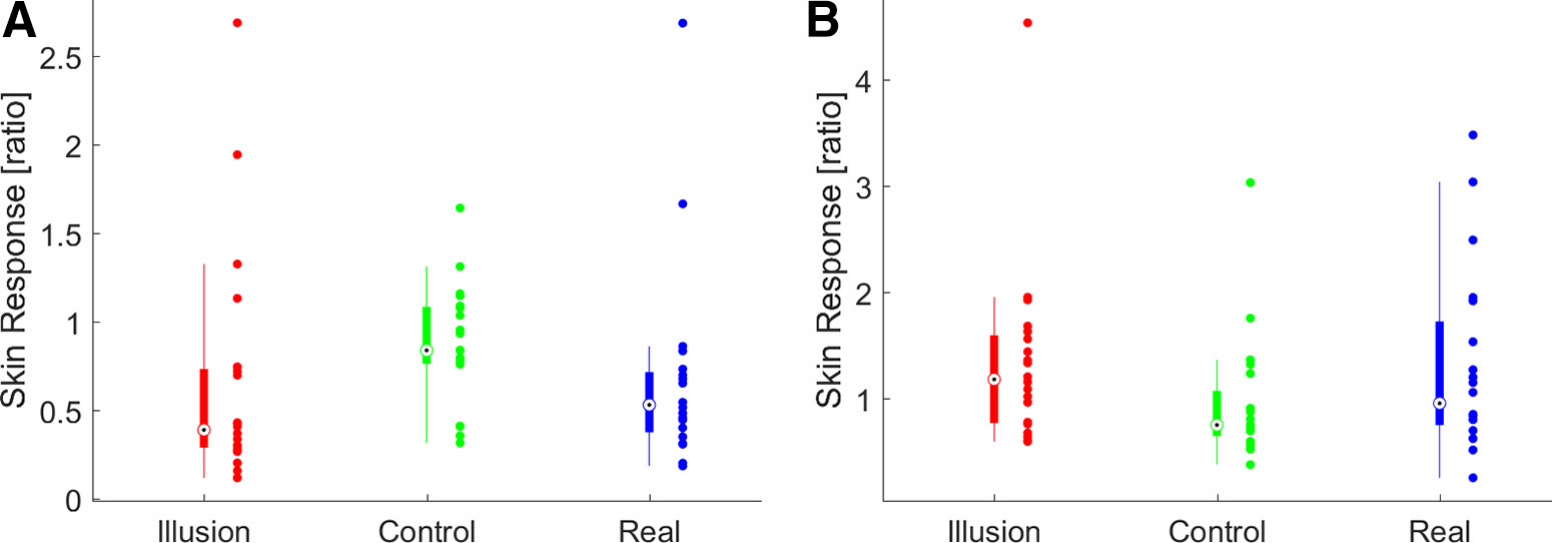
Changes in skin conductance associated with the illusion. Boxplots show skin responses for the two time windows: (***A***) around the illusion onset ([−3, 0 s] to [−0, +3 s]) and (***B***) before the reported onset of the illusion ([−6, −3 s] to [−3, 0 s]. Skin responses were defined as the ratio of the conductance in the two windows of interest. Boxplots indicate the median (circle) and 25th and 75th percentiles (thick line). Dots indicate individual participants (*n* = 20).

**Figure 3.**
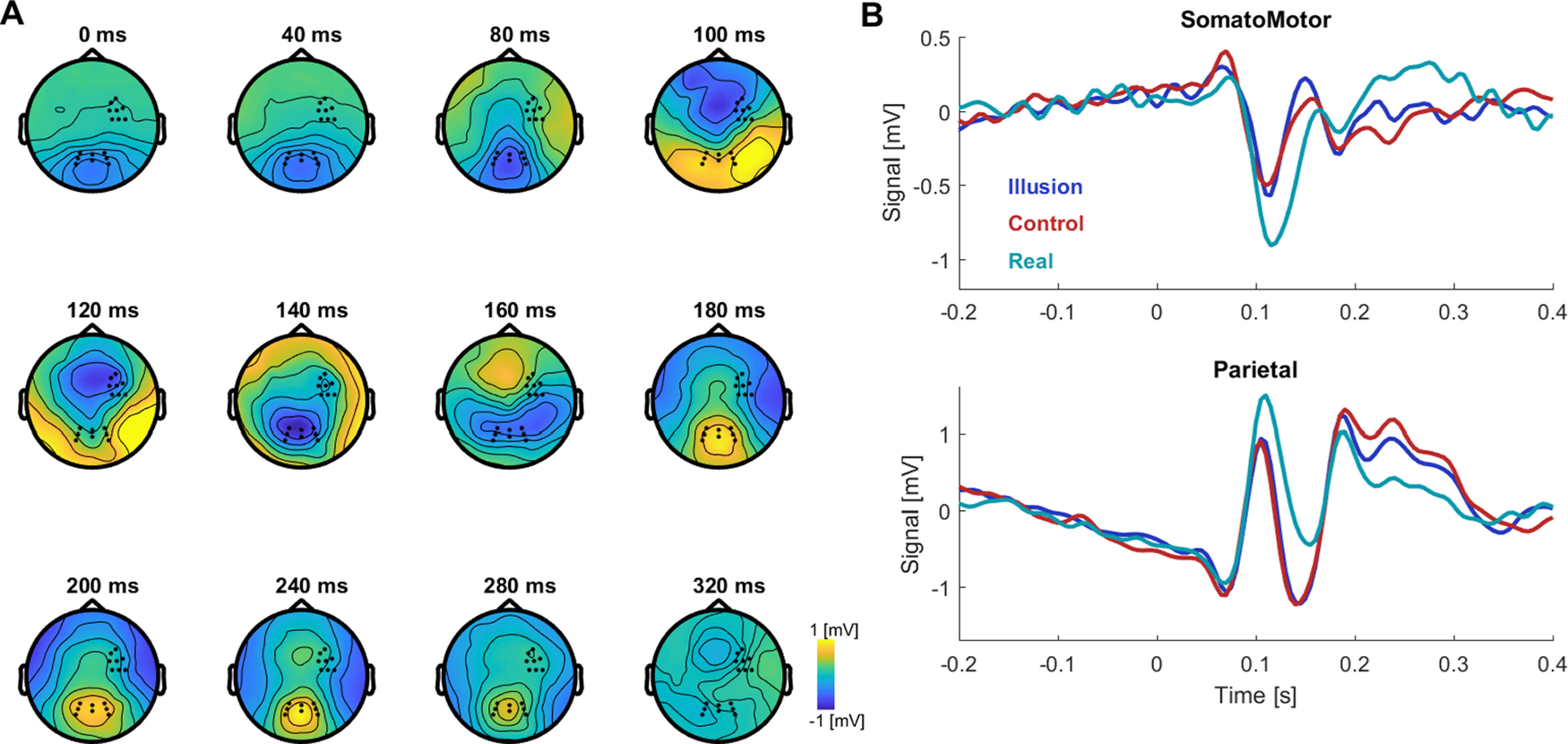
Illustration of evoked responses. ***A***, Average response across all experimental conditions shown as topographical time course aligned on the onset of the repetitive visuo-tactile stimuli at time 0 (*n* = 22). ***B***, Time courses for two regions of interest (the respective electrodes are indicated in the topographies in panel ***A***). Regions of interest were defined over right somato/motor electrodes based on their position in the EEG BioSemi cap and on previous studies that have reported effects related to the rubber hand illusion at similar locations (Press et al., 2008; Zeller et al., 2015; Rao and Kayser, 2017) and for comparison over parietal electrodes. See Materials and Methods for the effective number of data epochs for each condition.

**Figure 4.**
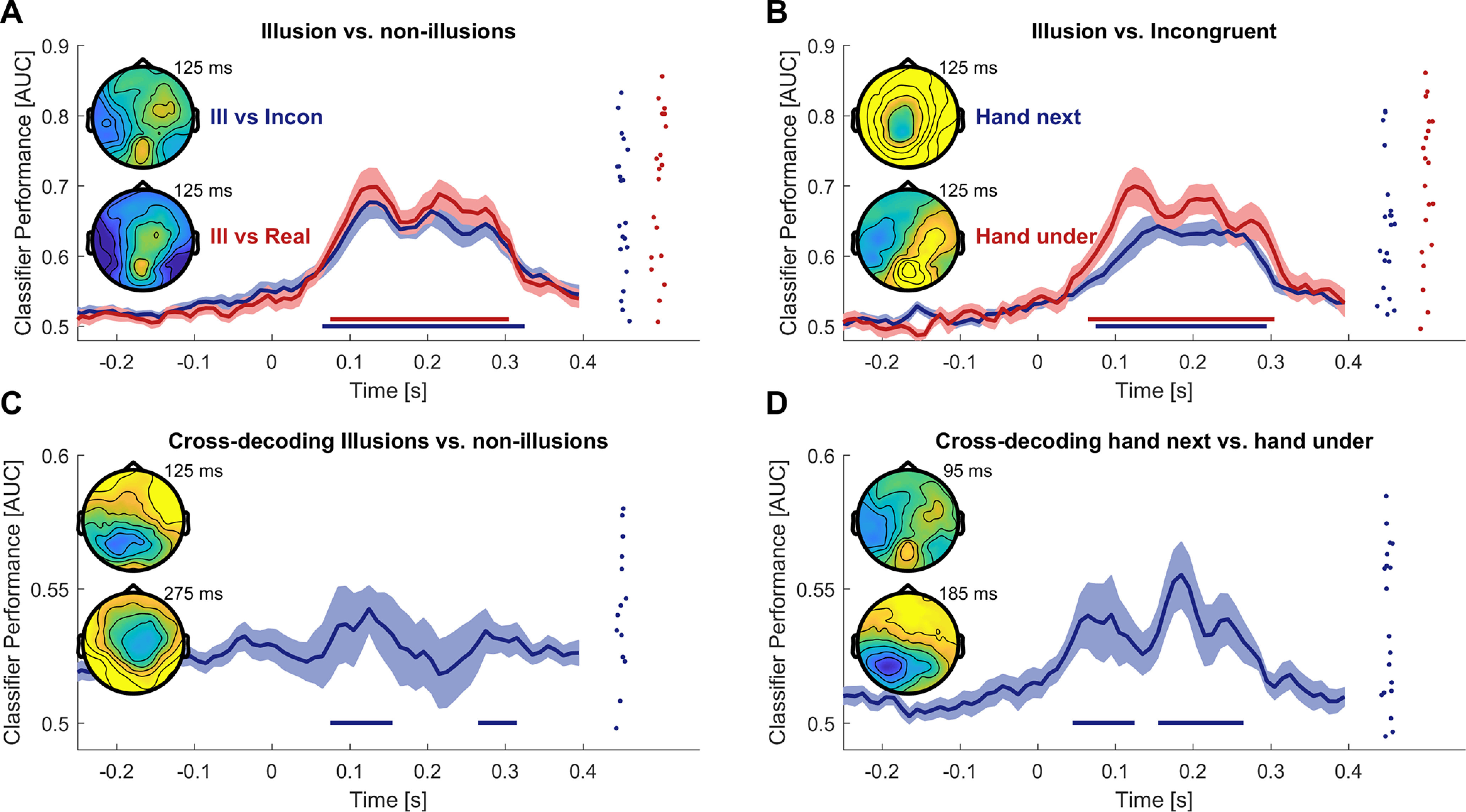
Classification analysis reveals long-lasting activations associated with the illusion. ***A***, Contrasting evoked responses between Illusion and Incongruent conditions and between Illusion and the Real conditions using linear multivariate classification revealed significant classification performance for all comparisons. The time axis refers to the center of the time windows used for classification. Classifier performance was computed as the area under the receiver-operator characteristic (AUC). Thick curves indicate the mean and shaded areas indicate the SEM. Straight lines at the bottom indicate periods of significant performance (cluster-based permutation test corrected for multiple comparisons along time, at *p* < 0.01). Topographies indicated the group-averaged forward models (color scale −0.3 to 0.3), and dots indicate individual participant’s classifier performance at the time points of maximal classifier performance (as indicated besides the topographies). ***B***, Results for the classification between each individual Illusion condition and the respective Incongruent condition. ***C***, Cross-classification analysis between Illusion versus Incongruent and Illusion versus Real conditions, probing for activations that reliably differentiate Illusion and non-Illusion epochs across experimental conditions. ***D***, Cross-classification between each individual Illusion and its respective Incongruent condition, i.e., testing whether activity patterns differentiating Illusion and Incongruent “hand next” conditions also differentiate these for “hand under,” and vice versa. The individual data (*n* = 22) are from time points 125 ms (***A–C***) and 95 ms (***D***), respectively.

**Figure 5.**
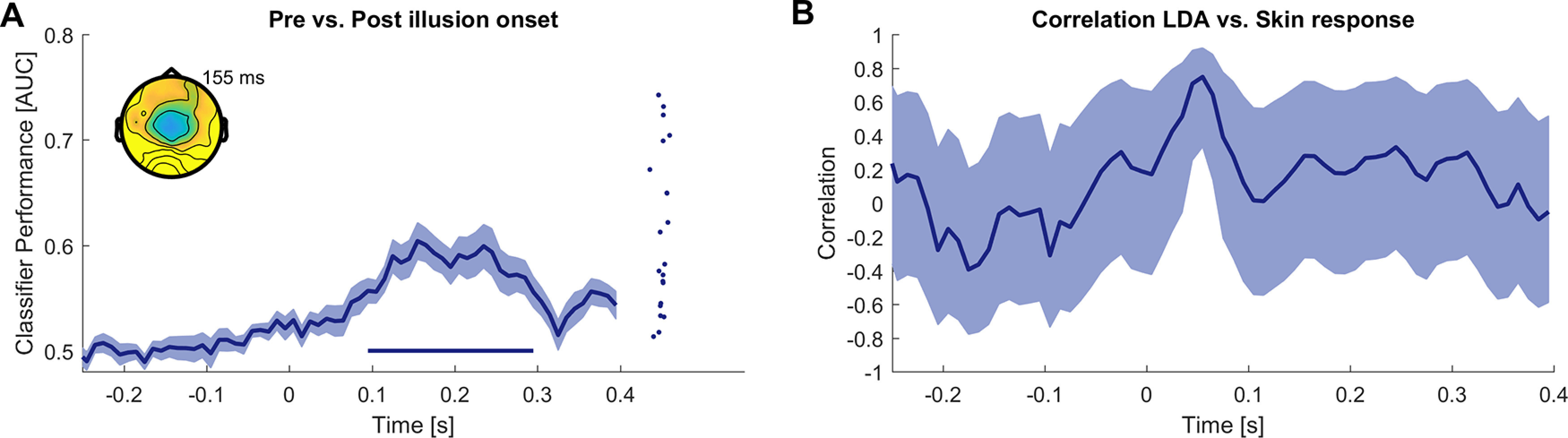
Within-trial illusion correlates and probing for an association with skin responses. ***A***, Classification of EEG activity between epochs before the illusion onset and the epochs after the illusion onset within the Illusion trials. Thick curves indicate the mean and shaded areas indicate the standard errors of the mean. Straight lines at the bottom indicate periods of significant classification performance (*n* = 22; cluster-based permutation test correcting for multiple comparisons along time, at *p* < 0.01). Scalp topographies show the classifier forward model and dots the individual classifier performance at the time point of maximal performance (color-scale −0.3 to 0.3). ***B***, Results of a correlation analysis between the classification performance for Illusion versus Incongruent epochs and the difference in skin response between these conditions. The solid line indicates the group-level Pearson correlation, the shaded area the 95% confidence interval (*n* = 20).

Importantly, by combining the signals from all electrodes into the multivariate classification process, this analysis does not make the assumption that the condition-wise spatial configuration of the relevant activity is the same across participants. Rather, it allows experimental conditions to differ reliably within each participant by a specific spatial pattern of activity, but this pattern can differ between participants. The analysis implemented here effectively asks whether at any moment during the data epoch one can reliably differentiate two conditions in each individual participant such that the group-level classification performance is significantly different from chance, see below for the statistical details (Cichy and Teng, 2017; Grootswagers et al., 2017). This classification analysis was used to test for EEG responses differentiating: (1) the Illusion versus Incongruent epochs (across both hand positions, Fig. 4*A*; and separately for each hand position, Fig. 4*B*); (2) the Illusion versus Real epochs (Fig. 4*A*); (3) and for activity differentiating those epochs within the Illusion trial before the onset of the illusory state (hence before participant’s button press) versus those during the illusion (i.e., after the button press; Fig. 5*A*).

To directly test whether the neurophysiological signatures discriminating the Illusion and Incongruent conditions are the same as those also differentiating the Illusion and Real conditions, we used cross-classification (Kaplan et al., 2015). For this, we trained the classifier on half the Illusion and half the Incongruent trials and then applied classifier weights to differentiate the remaining half of the Illusion and Real trials, repeating this procedure 50 times. Cross-classification was quantified using the AUC and computed for both directions: training on Illusion versus Incongruent and testing on Illusion versus Real, and training on Illusion versus Real and testing on Illusion versus Incongruent. The respective AUC values for each participant and time point were averaged over both directions and repeats of the calculation (Fig. 4*C*). The same cross-classification analysis was also used to test whether the neurophysiological signatures discriminating Illusion and Incongruent for the “hand next” arrangement generalize to the “hand under” arrangement (Fig. 4*D*), and to test whether classifiers trained on the discrimination of Illusion versus Incongruent also generalize to the discrimination of the epochs before the Illusion versus those during the Illusion (reported in text). A summary of all classification results is also provided in Table 2.

**Table 2:** Classification results

	*p*-value	AUC_sum	Time interval	Max AUC	Time of max AUC (s)
Illusion vs Incongruent	<0.001	3.68	[0.065–0.325]	0.68	0.125
Illusion vs Real	<0.001	3.90	[0.075–0.305]	0.70	0.135
Illusion vs Incon. “next”	<0.001	3.88	[0.07–0.305]	0.70	0.125
Illusion vs Incon. “under”	<0.001	2.97	[0.08–0.305]	0.67	0.135
Within Illusion trials	<0.001	3.43	[0.03–0.300]	0.65	0.13
Cross: (Ill vs Incon.) to (Ill vs Real)	=0.001 0.011	0.33 0.19	[0.075–0.155] [0.265–0.315]	0.54 0.53	0.125 0.275
Cross: (Ill vs Incon. “next”) to (Ill vs Incon. “under”)	0.002 <0.001	0.32 0.48	[0.45–0.125] [0.155–0.265]	0.54 0.56	0.095 0.185
Cross: (Ill vs Incon.) to (Within Illusion trials)	-	-	-	0.52	0.30

The table lists all pairwise classification and cross-classification (Cross:) results reported in the study. For each classifier we report the *p*-value of the significant cluster(s), the cluster statistics (AUC_sum), the time interval of the significant cluster(s), the maximal AUC value, and the time point of this.

#### Recording of skin conductance signals

Skin conductance was continuously recorded using a Neulog GSR logger NUL-217 sensor with Ag/AgCl electrodes were placed on the palmar sites of the middle and ring finger of the participant’s left hand. GSR measurements were recorded in micro-Siemens and at a sampling rate of 100 Hz. For offline analysis the data were resampled to 50 Hz, bandpass filtered between 0.5 and 2 Hz (third-order Butterworth filter). Trials were visually inspected and few trials with artefacts were manually removed. For a few participants (*n* = 2) the skin conductance data were incomplete, as the software had crashed during recording. Hence the effective participant sample for EEG (*n* = 22) and skin responses (*n* = 20) differed.

#### Analysis of skin conductance data

We opted for an analysis that directly contrasts the skin conductance in two adjacent time windows of 3-s duration. These windows were chosen around the key event of interest: the trial-specific onset of the illusion indicated by participants pressing the response button. The duration of the analysis window was chosen as a compromise between the typical latency of changes in skin conductance after an event (Boucsein et al., 2012; Sjouwerman and Lonsdorf, 2019) and the reaction times at which participants started feeling the illusions. To quantify the change in skin conductance (termed “skin response”) induced by the illusion onset we computed the standard deviation of the signal in two adjacent windows around the illusion onset (e.g., [−3, 0 s] and [−0, +3 s] with 0 being the Illusion onset time) and computed their ratio. In one analysis we computed the ratio between the window immediately following the illusion onset ([0, +3 s]) and the immediately preceding window ([−3, 0 s]). In a second analysis, we focused on the ratio of the two windows immediately before the illusion onset (hence [−3, 0 s] and [−6, −3 s]). To calculate the standard deviation (and then, their ratio) for Incongruent and Real conditions, we used trial-number-specific reaction times obtained from the Illusion trial with that respective number as surrogate time points to define the corresponding windows.

### Statistics

Statistical testing of the classification performance (AUC values) relied on a randomization approach and cluster-based permutation procedures to control for multiple comparisons (Nichols and Holmes, 2002; Maris and Oostenveld, 2007). Following recommendations in the literature we implemented a group-level random-effects inference on the null hypothesis of no experimental effect, which here corresponds to classification performance being around chance (Cichy and Teng, 2017; Grootswagers et al., 2017). In general, this is obtained by permuting the participant-wise condition labels of the test of interest (Maris and Oostenveld, 2007). Here, the randomization distribution was obtained by shuffling the sign of the true single-participant effects (i.e., the sign of the chance-level corrected AUC values) based on which we derived a distribution of expected group-level effect of no systematic classification performance across participants. We relied on 5000 randomizations for each test, used a cluster-forming threshold corresponding *p* < 0.01 (i.e., using the 99th percentile of the full distribution of randomized AUC values), applied spatial clustering based on a minimal cluster size of 3, and used the sum of AUC values within each cluster as cluster-wise test statistics. For significant clusters we report the cluster statistics (summed AUC value) and the peak classification performance (max AUC). The correlation between classification performance and differences in skin responses between Illusion and Incongruent conditions was based on a between-participant Pearson correlation, and statistical significance was established again using a cluster-based permutation approach, using a first-level threshold of *p* < 0.01 and by shuffling the assignment of AUC values and skin responses across participants. To compare the median latencies of illusion onsets between conditions and to compare the skin responses between pairs of conditions we used Wilcoxon signed-rank tests. Effect sizes for this test were obtained using the point-biserial correlation (denoted r_rb_; Kerby, 2014).

## Results

### Illusion onset times

We compared the mean onset times of the illusory states between the two Illusion conditions. The onsets in the “hand next” condition occurred on average after 51 ± 7.7 s (*n* = 22; mean ± SD; median: 42 s) and significantly later compared with the “hand under” condition (34 ± 5.9 s, mean ± SD; median: 30 s; Wilcoxon signed-rank test *Z* = 2.13, *p* = 0.03, r_rb_ = 0.45). Hence, the hand under arrangement required less time to induce the illusion, despite the physical distance between the participant’s and the rubber hand being the same in both configurations. We note that these latencies are comparable to previous work using a similar experimental setup (Rao and Kayser, 2017).

### Skin conductance

We implemented two explorative analyses focusing on changes in skin conductance emerging either in parallel with the participant’s overt response of feeling the illusion (button press), or before this. The analysis centered on the reported illusion onset revealed no significant differences in skin response between Illusion and Incongruent conditions (*n* = 20, Wilcoxon sign-rank test, *Z* = −2.13, *p*_corr_ = 0.100, r_rb_ = −0.54; Fig. 2, left) or between Illusion and Real conditions (*Z* = −0.6, *p*_corr_ = 0.82 r_rb_ = −0.15, with *p*-values corrected across tests using the Benjamini–Hochberg procedure). The analysis focusing on the epochs before the onset revealed significantly stronger skin responses during the Illusion compared with the Incongruent condition (*Z* = 2.99, *p*_corr_ = 0.003, r_rb_ = 0.76; Fig. 2, right), but not between the Illusion and Real conditions (*Z* = 0.26, *p*_corr_ = 0.79, r_rb_ = −0.06).

### Evoked responses differ between Illusion and control conditions at multiple latencies

We then asked whether and how EEG activity differs between epochs in which participants feel the illusion (i.e., the illusory state) and the different non-Illusion conditions. Specifically, we focused on the electrophysiological responses evoked by the repetitive visuo-tactile stimulus as a signature of the cerebral processing of the repetitive visuo-tactile stimuli used to induce the illusion. To illustrate these, Figure 3 shows the trial-averaged and participant-averaged evoked responses. This suggests that differences between the three main experimental conditions may exist at various points in time and for a prolonged time.

To systematically probe for differences in the spatiotemporal patterns of evoked responses between conditions, we relied on a multivariate classification framework. Group-level classification performance of Illusion versus Incongruent epochs was significant from 0.065 to 0.325 s (*n* = 22, cluster-based permutation test correcting for multiple comparisons along time, *p* < 0.001, AUC_sum = 3.68, max AUC = 0.68 at 0.125 s; Fig. 4*A*; see also Table 2 for an overview over all classification results). In a subsequent analysis we confirmed that the difference between Illusion and Incongruent conditions prevailed for both relative positions of the artificial and real hands: classification of each Illusion versus the respective Incongruent condition was significant in similar time windows (“hand next”: from 0.075 to 0.305 s, *p* < 0.001, AUC_sum = 3.88, max AUC = 0.70 at 0.125 s; “hand under”: from 0.08 to 0.305 s, *p* < 0.001, AUC_sum = 2.97, max AUC = 0.67 at 0.135 s; Fig. 4*B*). Classification of the Illusion versus the Real condition was significant in a similar long window (cluster from 0.075 to 0.305 s, *p* < 0.001, AUC_sum = 3.90, max AUC = 0.70 at 0.135 s; Fig. 4*A*).

We then asked whether the representational differences between the Illusion and Incongruent conditions and between Illusion and Real conditions arise from the same neurophysiological processes and hence have a similar spatial configuration within each participant. To address this question we relied on the concept of cross-classification (Kaplan et al., 2015; Cichy and Teng, 2017). Practically, this was implemented by training the classifier to discriminate between one pair of conditions (e.g., Illusion vs Incongruent) and testing this on another pair (e.g., Illusion vs Real) using non-overlapping sets of trials. The cross-classification performance was significant in two clusters (between 0.075 and 0.155 s, *p* = 0.001, AUC_sum = 0.33, max AUC = 0.54 at 0.125 s; and between 0.265 and 0.315 s, *p* = 0.011, AUC_sum = 0.19, max AUC = 0.53 at 0.275 s; Fig. 4*C*), suggesting that during these times the Illusion epochs are distinguished from both types of non-Illusion epochs by a consistent pattern of evoked response. We also used cross-classification to confirm that the neurophysiological processes differentiating the Illusion and the Incongruent for the “hand next” conditions shared a similar spatial configuration compared with those differentiating these conditions during the “hand under” configuration: cross-classification was significant for most time points (between 0.045 and 0.125 s, *p* = 0.002, AUC_sum = 0.32; max AUC= 0.54 at 0.095 s; and between 0.155 and 0.265 s, *p* < 0.001, AUC_sum = 0.48; max AUC= 0.56 at 0.185 s; Fig. 4*D*). Together, these results suggest that the neurophysiological processes differentiating the Illusion from the non-Illusion conditions fall into two types: one that generalizes across non-Illusion conditions (Incongruent and Real), and one for which activity selectively differentiates the Illusion from just one of these control conditions.

### Evoked responses differ within Illusion trials according to the subjective state

Comparisons between Illusion and control conditions are performed by comparing epochs from distinct experimental trials provided at different times during the experiment. Hence these may differ not only in the subjective illusory state. In a separate analysis, we investigated a contrast to isolate correlates of the illusory state in which we aimed to render the comparison of illusion and non-illusion-related activity more specific; for this, we directly contrasted epochs characterized by the illusory state and those reflecting non-Illusion epochs within the same experimental Illusion trials (Ehrsson et al., 2004; Guterstam et al., 2019). We split the epochs from the Illusion trials into those before the illusion onset and those during illusory state based on participants’ responses indicating the onset of the illusory state. A classification contrasting these epochs was significant (*n* = 22, cluster from 0.095 to 0.300 s, *p* < 0.001, AUC_sum = 1.71, max AUC = 0.60 at 0.155 s; Fig. 5*A*).

We then asked whether the activity patterns differentiating these pre-Illusion and Illusion epochs within the same experimental trials are similar to those differentiating the Illusion and Incongruent epochs between trials (compare Fig. 4*A*). To address this, we trained a classifier on the pre-Illusion versus Illusion classification and tested whether this generalizes to the discrimination of Illusion versus Incongruent epochs: this cross-classification analysis did not yield any significance (cluster-based permutation tests, no clusters at the respective criteria; max AUC = 0.52 at 0.300 s).

### Neurophysiological signatures of the illusion and skin responses

Given that the illusory state was associated with changes in EEG responses and with changes in skin conductance, we asked whether the respective effects were correlated across participants. To test this, we computed the correlation between the LDA classifier performance in discriminating Illusion and Incongruent epochs as a measure of the participant-wise illusion-effect in the EEG signal and the differences of skin responses in Illusion and the Incongruent conditions. We focused on the Illusion versus Incongruent comparison as for this specific comparison the skin responses had revealed a significant effect (compare Fig. 2). We found a no significant correlation (*n* = 20 participants with usable skin responses; cluster-based permutation statistics; cluster from 0.035 to 0.065 s, trending *p* = 0.061, sum = 2.61 peak value 0.75 and peak time 0.055 s).

## Discussion

We investigated the neurophysiological correlates of the RHI using a multivariate (cross-)classification framework and asked whether and when evoked responses differentiate the illusory state reliably from multiple and different control conditions used to isolate correlates of the illusory state. Thereby the present study aimed to make both methodological and conceptual advances on our understanding of the neurophysiological signatures of body illusions such as the rubber hand, as discussed below. We found that EEG responses differed between Illusion and non-Illusion epochs starting at latencies of ∼65 ms following stimulation onset and did so over a prolonged time. Importantly, around two time points, 125 and 275 ms, the illusory state was reliably differentiated from multiple non-Illusion conditions by a common spatial pattern of evoked responses in each participant, pointing to neurophysiological processes possibly specifically associated with the illusory state.

### Correlates of the illusory state in neurophysiological responses

A number of studies have aimed to understand the EEG-derived correlates of the RHI by investigating the responses evoked by the illusion-inducing stimuli (Press et al., 2008; Cardini et al., 2011; Zeller et al., 2015; Rao and Kayser, 2017; Guterstam et al., 2019). This approach rests on the idea that the neurophysiological signatures in the evoked response can reliably index the relevant neurophysiological processes differentiating the illusory state from suitable control conditions. However, these previous studies provide diverging results: while some report a modulation of evoked responses at early (55 ms) latencies (Otsuru et al., 2014; Zeller et al., 2015) others report effects around 140–200 ms (Press et al., 2008) or even later latencies such as between 300 and 450 ms (Rao and Kayser, 2017). Furthermore, while some studies suggest an enhanced evoked response during the illusory state (Press et al., 2008; Aspell et al., 2012), others rather point to a suppression (Zeller et al., 2015), or found no significant difference (Pyasik et al., 2021). Importantly, most studies effectively mapped group-level activations and made the implicit assumption that the neurophysiological sources differentiating Illusion and non-Illusion conditions have the same spatial (electrode-wise) configuration across participants, which may not be valid. In support of this notion, one study emphasized the distributed nature of the activity related to bodily illusions whereby distributed patterns may better reflect the illusory state rather than localized effects (Aspell et al., 2012). To shift the focus from group-level activations to a representational framework (Kriegeskorte et al., 2006; Hebart and Baker, 2018), we here exploited a multivariate classification approach. This allowed us to relax the assumption of a common spatial pattern of illusion-related evoked response across participants, providing a methodological shift from previous studies. Importantly, it also allowed us to directly test within each participant at which time point the relevant neurophysiological signatures generalize between conditions, such as the Incongruent and Real conditions, or horizontal and vertical arrangements of real and artificial hands. Along this conceptual level, our results show that evoked responses differ reliably between Illusion and non-Illusion epochs over prolonged periods (see Table 2 for an overview). This result effectively allows consolidating the previous work, as the present data support multiple of the previously reported illusion correlates.

The earliest differences between Illusion and non-Illusion epochs emerged around 65 ms from stimulus onset. This finding corroborates previous studies, which have pointed to such early illusion-correlates as possibly arising from somatosensory cortices (Zeller et al., 2015; Sakamoto and Ifuku, 2021). Still, these did not test whether these illusion-correlates generalize between control conditions. Addressing this, our cross-classification analysis suggests that at these latencies illusion-related neurophysiological signatures indeed generalize across distinct non-Illusion conditions.

Both the decoding and cross-classification performances peaked around 120–130 ms. The classifier forward models around these time points (compare Fig. 4) revealed a differential contribution of bilateral fronto-parietal sensors, as opposed to for example a lateralization of the relevant processes. A previous study using a full body illusion using a has attributed activations around these latencies to higher somatosensory and temporo-parietal regions involved in the integration of visual and tactile inputs about body position (Aspell et al., 2012).

Between 150 and 250 ms, the illusion-related processes generalized across hand positions (Illusion vs Incongruent contrast) but did not generalize to the Real condition, despite other activity patterns differentiating Illusion and Real conditions around the same time. Our interpretation is that activity during this time window reflects two kinds of processes: one that detects the spatiotemporal mismatch of the multisensory information between the visual and somatosensory inputs and gives rise to the difference between Illusion and Incongruent conditions. Another is sensitive to the embodied nature of the seen hand and whether the control object is posed in a realistic body position, giving rise to the difference between Illusion and Real conditions (Otsuru et al., 2014; Zeller et al., 2015).

Lastly, from ∼265 to 315 ms, the illusory state could again be reliably differentiated from all control conditions by a common activity pattern in each participant. Previous work has attributed potential illusion-correlates at these latencies to higher parietal and frontal regions, which have been implied in the formation of the illusory state based on multiple lines of evidence (Ehrsson et al., 2004, 2005; Petkova et al., 2011; Blanke, 2012; Bekrater-Bodmann et al., 2014). Activity at these latencies may in principle reflect a number of factors, such as spatial attention (Press et al., 2008; Rao and Kayser, 2017), bodily self-detection (Galigani et al., 2021) and high-level processes pertaining to multisensory causal inference (Samad et al., 2015; Ehrsson and Chancel, 2019; Fang et al., 2019), but again previous work has not shown that the neurophysiological representations differentiating the illusory state generalize across multiple control conditions. A parsimonious explanation may be that around these latencies parietal and premotor regions combine the available visual, tactile and proprioceptive signals with preexisting body representations to give rise to the illusory ownership feeling (Press et al., 2008; Lippert et al., 2013; Quinn et al., 2014).

### The impact of methodological details on outcomes in studies on the RHI

A central issue with studies on the RHI or similar body illusions is that the experimental contrasts generated to isolate the neural correlates of the illusory state are not specific to just this subjective state. This is because the data obtained during the experimental conditions inducing the illusion and those during the respective control conditions differ along multiple dimensions. For example, the data obtained during the Incongruent control condition and the Illusion condition differ in that participants were experiencing the illusion only in the Illusion condition, but differ also in the orientation of the rubber hand (Kalckert and Ehrsson, 2012, 2014; Rao and Kayser, 2017). Similarly, the Illusion and the Real conditions differ in the nature of the viewed stimulated hand (Zeller et al., 2015, 2016; Rao and Kayser, 2017), and in some other studies the conditions inducing the illusion and serving as control differed in the temporal pattern of the applied sensory stimuli (Botvinick and Cohen, 1998; Bekrater-Bodmann et al., 2012; Riemer et al., 2015). As a result, it is difficult to associate the neurophysiological correlates of the illusory state obtained in any individual and pairwise statistical comparison with a unique aspect of this paradigm (Rao and Kayser, 2017). In a conceptual advance to overcome this problem, we combined multiple control conditions with multivariate cross-classification, which allowed us to directly probe which neurophysiological processes consistently differentiate the illusory state from more than one non-Illusion condition.

First, we employed two spatial configurations of participants and rubber hands for the Illusion conditions, by displacing the rubber and real hands either in the horizontal or vertical planes, while keeping their physical distance the same. Our results show that the hand under arrangement required less time to induce the illusion, in line with studies reporting stronger illusory precepts for the vertical set-up based on questionnaire scores (Bekrater-Bodmann et al., 2012) and with the general idea that distances in the horizontal and vertical plane are often judged differently (Loomis and Philbeck, 1999). Importantly, the neurophysiological signatures differentiating the Illusion and Incongruent conditions generalized across the precise spatial arrangement of hands at many time points, hence are independent of the spatial plane in which the illusion is induced. Second, we employed both a rotated rubber hand and participant’s own hand to generate control conditions not inducing the illusion (Zeller et al., 2015, 2016; Rao and Kayser, 2017). Neurophysiological signatures that reliably differentiate the illusory state from both control conditions, e.g., around 120–130 ms in our data, should hence be insensitive to the precise nature of the second hand in sight, or the precise position of the second hand relative to the own body. However, whether this renders the respective neurophysiological correlates specific to only the subjective illusory state still remains unclear, and future work could combine the multivariate approach with additional illusion configurations or other non-illusion control conditions.

In an attempt to overcome this conundrum around comparing distinct experimental configuration, we directly contrasted activity within the Illusion trials between epochs before and while participants were feeling the illusion. This contrast pertains only to the Illusion configuration and allows a comparison of EEG responses obtained during the very same sensory input. Still, one may argue that also this comparison is potentially confounded by additional factors, such as participant’s task set (being ready to report the illusion onset before this, and having no task after the onset) or adaptation effects because of the repetitive stimulation of the somatosensory system over prolonged time (McLaughlin and Kelly, 1993). The cross-decoding results suggest that the neurophysiological processes differentiating the Illusion condition from both Incongruent and Real conditions may not allow differentiating the epochs before and following the onset of the illusory state in the Illusion trials, possibly for the above-mentioned reasons. This leaves it for future studies to probe whether there are indeed neurophysiological signatures of the rubber hand illusion that are genuinely specific to the illusory state and not confounded by additional factors.

The present study was based on a multivariate classification framework. This allowed us to relax the possibly invalid assumption made in previous studies that illusion-related activations have the same spatial configuration across participants. While this allowed us to derive time points at which brain activity possibly characterizes the illusory state, it makes it difficult to associate the underlying neurophysiological processes with specific brain regions. Future studies, for example relying on combined EEG-fMRI recordings, may capitalize on the present experimental and analytical approach to more precisely determine the brain regions correlating with or giving rise to the differences between Illusion and non-Illusion conditions. Such studies may also be able to determine whether processes differentiating epochs before and subsequent the illusion are indeed genuinely different from those differentiating, for example, Illusion from the Incongruent or the Real condition.

### Changes in skin conductance and body illusions

Bodily signals such as changes in skin conductance are frequently studied in the context of body illusions, yet their suitability as markers of the illusory state remain debated (Holle et al., 2011; Kammers et al., 2011; Holmes et al., 2012; Suzuki et al., 2013; Riemer et al., 2015; Crucianelli et al., 2018; Horváth et al., 2020). Often, a threat is applied to the embodied rubber hand, which induces changes in skin conductance compared with control conditions (Armel and Ramachandran, 2003; Ehrsson et al., 2007), although some studies also focused on skin conductance during the entire experimental trial (D’Alonzo et al., 2020). We here focused on the moments at which the illusory state emerged and asked whether this emergence is characterized by concomitant changes in skin conductance. Our data support a change in bodily state prior participants’ actual overt response of reporting the illusion, possibly because changes in arousal precede the subjective sensation of ownership. This finding seems in line with the general idea that changes in arousal are associated or can precede cognitive processes (Bechara et al., 1997; Critchley et al., 2000; Williams et al., 2000; Critchley, 2002; Dawson et al., 2011). The associated skin response was significant in comparison to the Incongruent but not when compared with the Real condition, reflecting a stronger skin response relative to the embodiment of the stimulated rubber hand, which does not happen in contrast to the stimulation of a real embodied hand. Furthermore, the skin responses were not correlated with the electrophysiological signatures, leaving it unclear whether and to what degree bodily and neurophysiological markers of the illusion reflect the same underlying processes (Holle et al., 2011; Kammers et al., 2011; Holmes et al., 2012; Riemer et al., 2015; Horváth et al., 2020).

In conclusion, by probing the correlates of the rubber hand illusion using a multivariate (cross-)classification framework we make a methodological step toward understanding those neurophysiological signatures of body illusions that are independent of the precise experimental configuration or statistical contrasts used to isolate these. Consolidating previous work, our results suggest that evoked responses around 125 and 275 ms may be interesting candidate time points for future studies, though they also highlight the interpretational difficulties when interpreting the correlates of subjective states such as body illusions.

## Synthesis

Reviewing Editor: Satu Palva, Helsingin yliopisto Decisions are customarily a result of the Reviewing Editor and the peer reviewers coming together and discussing their recommendations until a consensus is reached. When revisions are invited, a fact-based synthesis statement explaining their decision and outlining what is needed to prepare a revision will be listed below. The following reviewer(s) agreed to reveal their identity: NONE.

The manuscript describes a study that sets out to add further knowledge to our relatively limited understanding of the electrophysiological correlates of the rubber hand illusion (RHI). Using a 128 electrode EEG system and two different set-ups of the RHI, one with a vertical and one with a horizontal alignment of the fake and real hands, both with their own controls, that are then also compared to a “real only” condition. The study is heavily based on the design of previous work by Rao & Kayser (2017), albeit with a different theoretical premise. Novel contributions in this study are the inclusion of skin-conductance measures and a multivariate decoding approach. We welcome the merging of EEG data with physiological responses and the multivariate approach, but we currently have major concerns regarding the statistical implementation of the decoding approach, the interpretation of the findings, the lack of behavioral measures of the illusion, and the literature review outlined below.

Major comments:

1. The potentially most significant issue with the manuscript is the group-level analysis of classifier accuracy, which features very heavily throughout the paper. The authors employed permutation statistics to evaluate whether the classifier performance metric, AUC, is greater than chance. Across each of 5000 permutations, a random number of subjects had their AUC-values ‘reversed’; that is, the sign was changed. The idea of permutations in permutation tests is to simulate the null-hypothesis, which in this case would be that the data do not offer informational content to classify them into their respective conditions. Critically, if the null hypothesis were true, we would not expect a negative AUC, but one close to zero. If I do not misunderstand something, the permutation tests here are overly liberal because they put the observed values in relation to an unrealistic outcome that would not be expected even if the data were pure noise. Given that this approach has been applied extensively throughout the paper, this issue needs to be addressed.

2. It isn’t easy to know what is driving the pattern differences between different conditions. Are the differences related to the rubber hand illusion or other unspecific differences between conditions? For example, differences in seen orientation of the rubber hand (90 degrees or 0 degrees) could in itself create pattern differences? This issue is even more problematic because the authors use an analysis that allows different patterns between different combinations of electrodes to be present in different individuals. Not all participants experience the illusion, so there could be very different patterns in different people, which may have nothing to do with the actual illusion. Thus, it is difficult to know whether the results reflect important neurophysiological mechanisms that generalize to the population and are likely reproducible. Moreover, it is difficult to interpret the current results with respect to the previous literature and clearly explain what the critical novel findings are and what conceptual advances they bring in our understanding of the frontoparietal circuits that produce the rubber hand illusions. That there are differences in brain activity from a wide array of electrodes during the rubber hand illusion and various controls is a rather vague and expected finding; many previous studies have reported activity differences in premotor and posterior parietal areas during the illusion using a variety of neuroimaging and electrophysiological measures. The analysis method is novel and exciting, but in its current version, it is difficult to see the neuroscientific importance of the findings.

3. Another major limitation was that the rubber hand illusion was not quantified behaviorally in the different conditions and the different individuals. So, we do not know how strongly the participant felt the illusion in the different conditions and how differences in illusion strength across individuals related to the multivariate patterns.

4. How do the authors interpret the fact that SCR only discriminated illusion and incongruent conditions in epochs prior to participants’ subjective reports? What were the authors expecting, and why? This is briefly discussed in lines 477-482, but it is not sufficiently explicit. More critically, there were no significant differences in SCR between illusion and control conditions (lines 325-327), and the correlation between EEG illusion signatures and the SCR was NOT significant (line 436). On line 436, you write “close to significant,” and you talk about “trends” in the discussion, but this correlation is not significant and should not be interpreted as such. Overall, we are not impressed by the current SCR results and find them inclusive. Our suggestion is to tone down the importance of the SCR findings and not present them as one of the study’s major results or mention them in the abstract.

5. The cross-decoding approach employed very interesting and novel. However, the authors should discuss it further - the main point that they should unpack in the Discussion is whether this approach effectively isolates the neural signatures of the RHI and not the signatures associated with other perceptual low-level processes e.g., spatial discrimination of rubber-hand orientation. This could be addressed close to the following sections: (1) in lines 525-533 or (2) after the paragraph in lines 550-558.

6. The literature review is only of average quality. Some studies that do not investigate the rubber hand illusion are cited as if they are rubber hand illusion studies even though they investigate quite different illusion phenomena; some relevant previous human electrophysiological studies are not cited; some odd conclusions are made based on the previous literature. The authors need to read all the papers carefully they cite and make sure the citation is correct and relevant. See the various specific minor points below.

7. The comparison of the periods before the onset of the illusion in the synchronous condition to the period after onset is problematic because these periods differ in task-set. During the period before the participants have the task to detect illusion onset and prepare a response and this is not the case during the period after onset. Thus, task differences could confound the analysis of the results (both EEG and SCR), which should be considered.

8. A related point is the illusion onset times are surprisingly slow, 34 to 51 seconds, which is much slower than reported in previous studies where the onset times are more around 10 seconds or so (Ehrsson et al 2004; Lloyd 2007; Guterstam et al, 2013; Ehrsson et al 2005). Why do you think this is the case. Are the participants using an over-conservative criterion? Or is the illusion is much weaker in this study? Could this mean that you have illusion activity already in the period before reported illusion onset?

Minor comments:

9. Lines 40-41. Why are the neurophysiological correlates of the RHI controversial? What is finding controversial? Or do you mean we still know relatively little? Further, the papers you cite here are behavioral studies, which seems odd.

10. Lines 42-43. Check out the recent book chapter by Ehrsson 2020. A number of fMRI studies have reported consistent and reproducible activations in premotor cortex, posterior parietal cortex, lateral occipital cortex, and cerebellum related to the rubber hand illusion. The three references you give are good, but there are more, e.g., Gentile et al 2013 J Neuroscience and Limanowski et al, 2016 J Neurosci for example. And why do you say “body illusions” and not rubber hand illusion and similar full-body ownership illusions with mannequins viewed from the first-person perspective?

11. Lines 52. Aspell et al 2021 does not investigate the rubber hand illusion or similar body ownership illusion but uses a quite different paradigm where the body is viewed from a distance from a third-person perspective (see discussion in Petkova et al 2011 Frontiers or Preston et al 2015 PLOS ONE). I am not sure how relevant those EEG findings are for the current paper when the paradigms are so different.

12. At the end of page 3 and in the discussion, I miss a citation to the relevant human electrophysiological study by Guterstam et 2019 Cerebral Cortex. This study observed no changes in S1 but in high gamma power in the posterior parietal cortex and premotor cortex associated with the illusion and effective connectivity between S1 and posterior parietal cortex. Moreover, Guterstam further directly compared the ECoG responses to fMRI data in the same individuals and found significant overlap in relevant areas.

13. Lines 70-71 This sentence reads too strong. Many studies have described interesting illusion-related responses in localized patterns. The “distributed approach” just provides a different perspective.

14. Lines 94. The reference to Critchley seems out of place here. The best threat-evoked SCR study is probably Gentile et al 2013 J Neuroscience (see his second experiment); you could consider citing this one instead.

15. Lines 88-89. Guterstam et al 2019 also compared neurophysiological responses before and after illusion onset. Ehrsson et al 2004 also conducted comparisons of periods but with fMRI.

16. How many trials were done per participant? How long did the experiment last? Total number of trials per condition and per participant? Were conditions balanced in the number of trials? How did they manage to address this in the MVPA?

17. Why did the authors decide to filter the EEG data using a low-pass filter of 0.6? This may be considered a bit too high. The authors could explain briefly why they think this is fine.

18. Lines 138-141. The asynchronous condition is a very effective control condition the eliminates or strongly suppresses the RHI. These statements are wrong and must be changed. The problem with the asynchronous condition in EEG studies with their high temporal resolution is that this condition can be seen as a series of alternating unisensory stimuli rather than a multisensory condition.

19. Lines 142-143. Just looking at the RHI without any dynamic stimulation typically does not elicit the RHI. See for example, Guterstam et al 2019 PLOS ONE (doi: 10.1371/journal.pone.0213265. The results of the Ferri et al 2013 study are somewhat odd and can probably not be replicated, in my opinion. It should be cited with caution.

20. “Data” is always plural; in line 144 it says, “our data suggests”.

21. Lines 182-184. Typically, two-thirds of the participants experience the RHI, not half as it is stated here. See, for example, Reader et al 2021 Frontiers (doi: 10.3389/fpsyg.2021.629590). You should double-check these citations.

22. Line 261 - Are Incongruent and Control not the same? If they are, please use the same term, especially in this context.

23. The participants pressed a button upon illusion onset - can the authors be certain that this response requirement did not influence the skin conductance response?

24. Why did the authors choose the segmentation from -400 to +400 ms? Figures only show either 200 ms or 250 ms baselines, which in the ERP figures do not have a mean of 0. Also, I wonder how the authors decided on such a short time window for the post-stimulus time window when the very similar Rao & Kayser study (2017) found effects up to 450 ms.

25. Why was the data filtered twice (first up to 90 Hz, then 40 Hz after segmentation)? Since multivariate approaches in time-series data have not been as popular as in fMRI, I wonder whether the filtering process here should receive some more consideration - do we lose potentially critical informational content if we filter too harshly?

26. On a related note, why did the authors resample the EEG signal at 200 Hz? While this may be considered okay for some slow ERPs (e.g. P3), it may not be ideal for faster EEG features. I do not consider this a critical issue, but it should be briefly addressed in the Methods section.

27. Why did the authors use 6-fold cross-validation? Why did they choose 6-fold and not 10-fold? Most visual studies, for example, use 10-fold cross-validation. If the authors had a rationale behind this decision, they should make it explicit in the Methods section, even if their rationale was not based on methodological or scientific reasons (e.g. maybe the authors simply wanted to reduce computing time).

28. Are unequal trial numbers not a problem for the decoding approach? It might give one condition an advantage over another - which would mean we would need to take a look at sensitivity/specificity.

29. Line 341: What are the “...relevant distant signals.”?

30. Line 342-343 - possibly word missing - unclear

31. In the ERP shown in Figure 3B, the voltage fluctuations associated with the “real” condition look much noisier in their baseline than in the other two conditions plotted there. Was the number of trials balanced between conditions in this analysis? If not, the authors should either argue why this is not a limitation for what they are testing there or rerun this analysis while keeping a matched number of trials per condition (e.g., undersampling).

32. Line 492 - We are getting confused with the number of different classifiers - how certain is it that the “processes” seen in this time-window are illusion-specific? Could they be control-specific, perhaps reflecting an attempt to consolidate the fact that the fake hand implicated in the visuotactile stimulus is placed in an awkward position? A more general suggestion we have for the paper would be to have a table with every classifier listed with its training and testing conditions that can easily be referred back to. At the moment it eventually just becomes difficult to follow.

33. Lines 486-487. The Aspell et al 2012 use a different paradigm, and higher somatosensory and TPJ responses are typically not seen in EEG and fMRI studies of the RHI . So consider deleting this.

34. There is a contraction in line 554: “can’t”.

35. Lines 507-508. You should cite causal inference studies of the rubber hand illusion here, and not only studies using audiovisual paradigms. For example, Samad et al 2015 PLOS ONE, Fang et al, 2019 PNAS, and Ehrsson and Chancel 2019 PNAS. You can check out see the recent preprint of Chancel and Wei Ji Ma (2021) https://doi.org/10.31219/osf.io/yh2z7.

36. Lines 529-530. We are afraid we have to disagree with this statement. It is not difficult to associate neurophysiological correlates of the rubber hand illusion by pairwise comparisons of conditions and univariate statistics. It is actually easier to interpret these previous results than the current more explorative “large scale” approach. Please reformulate to better focus on the advantage of your approach in a constructive manner.

37. Line 542. Please delete this sentence. Both the anatomically misaligned condition and the asynchronous condition are very effective in suppressing/eliminating the rubber hand illusion.

38. Is the real hand condition really a “control condition”? Should you not also expect similarities between the synchronous condition and the real hand condition? The participant experiences ownership of the hand in view in both the real hand and the rubber hand illusion conditions. I see the real hand condition as quite different from the anatomically misaligned control, for example.

39. Lines 564-566. I disagree with these statements. SCR can change independently of RHI, but you have the same problem in this study. To deal with this you need good controls, which previous studies have used (see for example Gentile et al 2013 J Neurosci or Guterstam et al 2015 Current Biology). Hand movements have not seen or registered in many previous threat-evoked SCR studies so this statement you have here is speculative and misleading.

40. Finally, I would suggest the authors add a brief conclusion section to their discussion because it is not sufficiently clear to the reader what the most important finding(s) are how they advance our understanding of the neurophysiological mechanisms of the illusion.

41. Figure 1 looks very poorly made (to put it mildly), the hands all have a frame around them, have different sizes, and lines of the boxes do not line up. Moreover, it is not clear from the figure whether the real hand was hidden at all.

42. Figure 2 also comes with unwanted frames around the plots.

43. Also, we noticed inconsistent capitalization of Figure/FIGURE X in the legends.

44. The final paragraph is confusing and contains speculations. It should be completely re-written to focus on your main findings and conceptual and methodological advance. Do not make conclusions based on non-significant results (trends).
